# Testicular length as an indicator of the onset of sperm production in alpacas under Swedish conditions

**DOI:** 10.1186/s13028-016-0191-x

**Published:** 2016-02-02

**Authors:** Maria Celina Abraham, Johanna Puhakka, Alejandro Ruete, Essraa Mohsen Al-Essawe, Kerstin de Verdier, Jane Margaret Morrell, Renée Båge

**Affiliations:** 1 Division of Reproduction, Department of Clinical Sciences, Swedish University of Agricultural Sciences, Box 7054, 75007 Uppsala, Sweden; 2 Department of Ecology, Swedish University of Agricultural Sciences, Box 7044, 75007 Uppsala, Sweden; 3 National Veterinary Institute, 75189 Uppsala, Sweden

**Keywords:** *Vicugna pacos*, Puberty, Animal husbandry, Statistical models, Decision making tools

## Abstract

**Background:**

The popularity of alpacas (*Vicugna pacos*) is increasing in Sweden as well as in other countries; however, knowledge about optimal management practices under Swedish conditions is still limited. The wide age range reported when the onset of puberty can occur, between 1 and 3 years of age, makes management decisions difficult and may be influenced by the conditions under which the alpacas are kept. The aim of this study was to find out when Swedish alpacas can be expected to start producing sperm, by using testicular length and body condition score as a more precise indirect indicator than age.

**Results:**

This study suggests that animals with a testicular length ≥3.8 cm would be producing sperm; however, if it is crucial to know that there is no sperm production for management purposes, the threshold level for testicular length used to differentiate between sperm-producing and non-sperm producing animals should be ≤1.6 cm instead. If only one variable is considered, testicular length appears to better than age alone to predict sperm production. Body condition score together with testicular length explains the individual onset of puberty and better guide management recommendations.

**Conclusions:**

Using a combination of these parameters (testicular length, body condition score and age) as a tool for decision making for alpaca husbandry under Swedish conditions is suggested.

## Background

Alpacas, llamas, guanacos and vicunas are South American camelids and members of the Camelidae family. Alpacas and llamas are domesticated, being kept as production animals (for fibre and meat production, as pack animals, etc.) as well as companion animals. In recent years, the international interest in breeding alpacas has increased together with the demand for more accurate information about their health care and animal husbandry. In Sweden, alpacas have become progressively more popular during the last decade; however, knowledge about optimal management practices and disease panorama under Swedish conditions is still limited [1, 2]. Alpacas present several physiological peculiarities compared with other domestic species. They are not considered to have a breeding season [3]. Like other camelids they are induced ovulators, with continuous waves of follicular growth throughout the year [4]. However the female may reject the male’s attempts to mate depending on the stage of follicular growth [3]. There are no reports of males showing seasonality in their willingness to mate.

It is known that nutritional status could influence the onset of puberty in other species [5] but according to Van Saun [6] studies on nutrition in alpacas and its relationship with reproduction are still lacking. Galloway [7] reported that there is a correlation between body size and testicular size, although the wide variation in testicular size suggests that other factors, probably genetic, are also important. Previous studies have shown that testicular size could be an indicator of sperm production and hence fertility, which is consistent with observations on the bull, ram and stallion [7, 8].

In addition to their physiology, they also have an anatomical difference. The prepuce has adherence to the glans penis until 2 or 3 years of age, making protrusion of the penis impossible in young males [9, 10]. The adherence disappears gradually when the animal matures, apparently in relation to testosterone levels [9, 11].

The detection of sperm production in males is an important factor in managing alpacas for several reasons, among them: (1) owners need to know when young males and females should be separated to avoid undesirable matings, (2) to use good males strategically in breeding programs, (3) to recommend when males should be castrated if they are not going to be used for breeding, (4) for research purposes, to know when it is the best moment to collect samples in order to access useful material, and (5) in a broader perspective, as an indicator of animal welfare.

Research in other countries indicates that male alpacas start producing sperm between 1 and 3 years of age [7, 9]. This wide range is probably influenced by the conditions under which alpacas are kept, and makes management decisions difficult. It is not known if the information generated in other countries, such as Perú, Australia and USA is applicable to Europe. Therefore, the aim of this study was to investigate with higher precision when Swedish alpacas can be expected to start producing sperm, by using the testicular length as an indirect indicator of the onset of sperm production. Two experiments were performed: Experiment 1 (on farm) to measure testicular size and Experiment 2 to determine the presence of sperm post castration or in cadavers. Whether this age could be affected by the body condition of the animal was also investigated.

## Methods

### Experiment 1

**Fig. 1:**
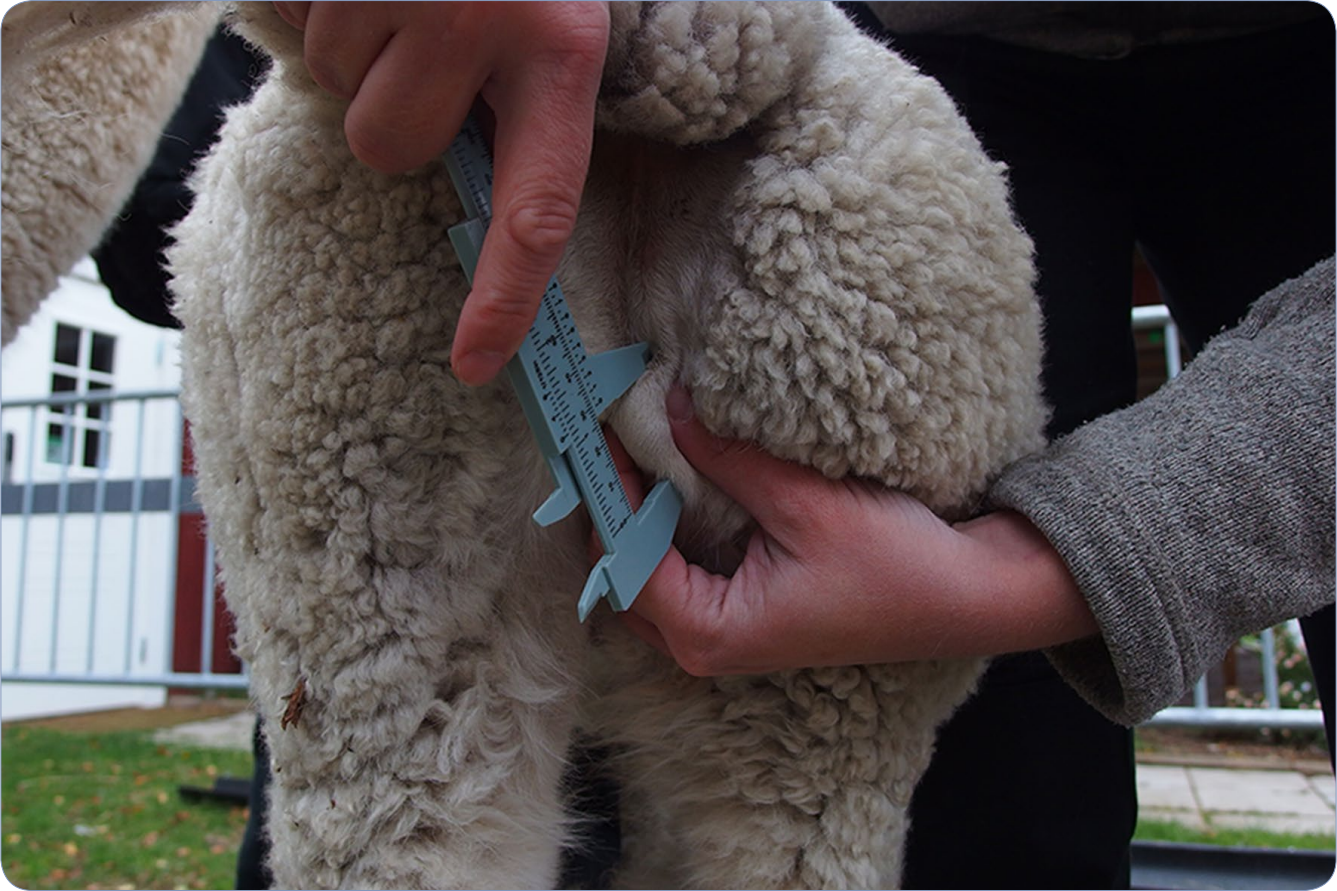
Measurement of testicles with a calliper

A total of 72 male alpacas, 13–48 months of age (median of 25.5 months), from 11 Swedish farms, were studied during September 2014. Farms were selected for convenience based on geographical distribution and number of animals. The length of the testicles was measured individually with a calliper (Biltema, Sweden) (Fig. 1).

Body condition was scored according to a standardized scale from 1–5, where 1 is emaciated and 5 is obese [12, 13].

Body condition scoring (BCS) was done by palpating over the central backbone near the last ribs. All of the testicle measurements and BCS were performed by the same operator.

### Experiment 2

#### Testicles from routine castrations

Twenty-two pairs of testicles were obtained from on-farm routine castrations of male alpacas on eight Swedish farms. The age of the animals ranged between 11 and 113 months (1–9 years) with a median age of 23 months. The study was conducted from May 2013 to April 2015.

The pairs of testicles were placed in plastic bags with phosphate-buffered saline solution (KV-laboratory, SLU) and were transported to the laboratory in styrofoam boxes containing a cold pack at 4 °C, where they arrived within 24–48 h of surgery.

#### Testicles from cadavers

Six pairs of testicles were obtained at necropsy from male alpacas originating from five Swedish farms. The age of the animals ranged between 16 and 96 months (1–8 years, median age of 42 months). The post mortem evaluations were conducted at Eurofins in Skara (Sweden) during October 2014–February 2015. The pairs of testicles were placed in plastic bags and were transported to the laboratory, where they arrived within a week of death. None of these animals died because of reproductive diseases.

### Procedure: measurement of testicles

After measuring the length of the testicles with a ruler, the tunica albuginea, connective tissues and blood vessels were removed and the epididymides were separated from the testes. The cauda epididymides were isolated, cut into 4–5 pieces and placed in a petri dish, in 1 ml of pre-warmed semen extender AndroMed (Minitüb, Tiefenbach, Germany) or INRA 96 (IMV Technologies, L′Aigle, France) for samples from castrations and in 1 ml of PBS for samples from necropsies. After 10 min of incubation at 37 °C in 5 % CO_2_ in an incubator, the presence of spermatozoa was verified using a phase contrast microscope Olympus BX 51 (Olympus, Japan) with 20 and 40× objectives.

## Statistical analysis

### Testicle size

The relationship between mean testicle length and the individual’s age was analysed. We assumed that the mean testicle length follows a normal distribution [*L*_*i*_ ~ Normal (*μ*_i_, σ)] for each individual *i* and increases over time given by:

$$\mu_{i} = \frac{\alpha }{{1 + e^{{ - \beta \cdot \left( {Age_{i} - \gamma } \right)}} }}$$

where *α* is the maximum mean testicular length, *β* and *γ* are free parameters determining the testicle length increment rate. The parameters where estimated under the Bayesian framework in JAGS 3.4 [14], because of its flexibility to estimate parameters uncertainty.

For samples obtained from experiment 1, we also tested the effect of body condition (*BCS*_*i*_) on the mean testicle length increment rate parameters (β, γ) as,

$$\mu_{i} = \frac{\alpha }{{1 + e^{{ - \beta \cdot \left( {Age_{i} - \gamma } \right) \cdot BCS_{i} \cdot \delta }} }}$$

using the mode of the posterior probability distribution of estimates for parameters β and letting the algorithms estimate α, γ and δ.

The models were evaluated for fit (using the deviance information criteria scores i.e. DIC, Bayesian analogues to the more common Akaike Information Criteria; [15]), and checking the posterior probability distribution.

### Sperm presence (logistic models)

We tested for the effect of age and mean testicle length on the presence of sperm in testicles from necropsies using logistic generalized linear models using the glm (family = “Binomial”) function on R v3.2 [16]. Model fit was evaluated through each model’s R^2^, comparing the model’s fit through AIC [17] and checking the statistical significance of the estimated parameter.

## Results

The distribution of mean testicular length by age groups in Experiment 1 is presented in Table 1. The distribution of mean testicular length by age groups and the presence of sperm in the testicles from castrations and cadavers (Experiment 2) are presented in Table 2.

**Table 1:** Mean testicular length and number of alpacas by age group (Experiment 1, in vivo measurements)

Mean length (cm)	12–23 months	24–35 months	36–48 months	Total
<3	8	0	0	8
3–3.9	14	8	0	22
>4	8	24	10	42
Total	30	32	10	72

**Table 2:** Mean testicular length, number of alpacas by age group and sperm presence (Experiment 2, testicles from castration n = 22; testicles from cadavers n = 6)

Mean length (cm)	Age^a^ (months)	Sperm presence (%)	
		Castration	Cadavers
<3	12–23	1/6 (17)	0/3 (0)
	24–35	1/4 (25)	–
	>36	1/1 (100)	–
3–3.9	12–23	3/4 (75)	–
	24–35	3/3 (100)	–
	>36	–	–
>4	12–23	–	–
	24–35	–	–
	>36	4/4 (100)	3/3 (100)

^a^Age at castration/necropsy

**Fig. 2:**
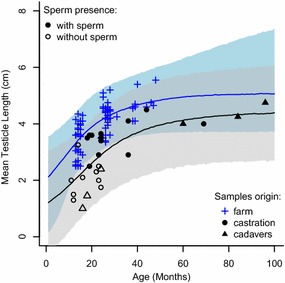
Length increment model for alpaca testicle length. Experiment 1, “farm”, n = 72; *solid line* (mean), and *dark grey polygon* (95 % CI); Experiment 2 (from castrations (n = 22) and cadavers (n = 6); *dashed line* (mean) and *light grey polygon* (95 % CI). Note: the measurements on living animals included the scrotal layers whereas organs obtained after castration or from cadavers did not

The testicle length increment model explained the variability in testicular length through time (Table 3). Estimates of increment rate parameters (β and γ) were similar between the experiments and the difference in maximum mean testicle length (α) is coherent with the expected difference in testicle length due to the collection methodology (Fig. 2). There was, however, large variation in maximum mean testicular length in samples from experiment 1 at greater ages, given the lack of data points in animals older than 50 months. The variability in samples from experiment 2 was generally higher than for experiment 1 but consistent throughout the age range (Fig. 2).

**Table 3:** Model fit (DIC and Delta DIC) and parameter estimates for testicle length of Swedish alpacas

Model	DIC	Delta DIC	α	β	γ	δ
Experiment 1						
Null	160.8	0	3.97 (3.8–4.14)			
Increment	117.3	43.5	4.96 (4.33–8.445)	0.078 (0.019–0.26)	5.89 (−0.87–29.77)	
Increment + BCS	116.7	0.6	4.75 (4.32–6.45)	0.078	5.4 (0.56–10.25)	0.35 (0.10–1.02)
Experiment 2						
Null	87.8	0	2.97 (2.55–3.39)			
Increment	66.9	20.9	4.43 (3.67–5.95)	0.069 (0.02–0.16)	15.47 (5.17–31.50)	

Values are (median 95 % CI in brackets). DIC scores are compared to the null model (including only an intercept parameter). For data from experiment 1, the model including body condition score (BCS) is compared to the increment model. Experiment 1, n = 72. Experiment 2, n = 28

*α* is the maximum mean testicular length

*β* and *γ* are free parameters determining the testicle length increment rate

δ BCS effect on increment rate

**Fig. 3:**
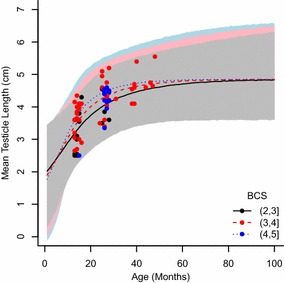
Length increment model for alpaca testicle length, incorporating age and body condition score (BCS). Testicle length measured in vivo (Experiment 1; n = 72); Circles represent individual values, lines represent means. *Black line* *and* *circle* = body condition score range from 2 to 3; *red line and circle* = body condition score range from 3 to 4, *blue line and circle* = body condition score range from 4 to 5. *Shaded area* indicates 95 % confidence interval

The BCS of the animals ranged between 2.5 and 5 with a median value of 4. A large variation between different ages was found. The length increment rate of testicles from experiment 1 was positively affected by BCS, while the maximum mean testicle length remained within the same range (Fig. 3).

**Fig. 4:**
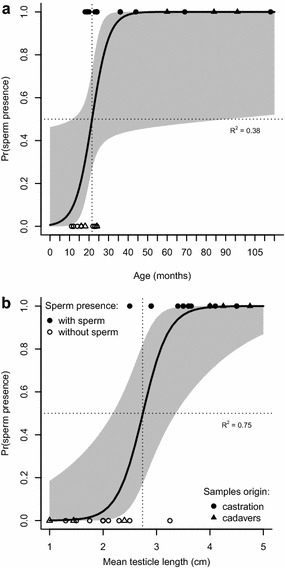
Probability of sperm presence in alpaca testicles. Samples from Experiment 2 (n = 28) explained by **a** age, and **b** mean testicle length. *Black circles* represent organs from castrations, *black triangles* represent organs from cadavers; *filled circles* represent organs containing sperm, *open circles* represent organs without sperm. Animals with testicular length ≥3.8 cm would be producing sperm (median probability of sperm presence is ≥0.99); and animals with testicular length ≤1.6 cm will not be producing sperm (median probability of sperm presence is ≥0.01)

There is high variability in mean testicle size 10 and 35 months (Figs. 2, 3). Indeed, the probability of sperm presence is better explained by mean testicular length (p = 0.01; R^2^ = 0.77; AIC = 14.97; Fig. 4a) than by the animals’ age (p = 0.047; R^2^ = 0.38, AIC = 27.87; Fig. 4b).

## Discussion

The wide range when the onset of puberty can occur, between 1 and 3 years of age, makes management decisions difficult and may be influenced by the conditions under which the alpacas are kept. The aim of this study was to find out if the same range could be expected in Europe (particularly in Sweden) as in other parts of the world. By using a model of testicular length that accounts for variability in age and BCS (Figs. 2 and 3), we tried to predict when alpacas can be expected to start producing sperm. As expected, we found that if only one parameter is considered, testicular length is better than age alone to estimate sperm production (Fig. 4) but that the inclusion of BCS and age together with testicular length gives better predictability than a single parameter. Therefore, we suggest using a combination of these parameters as a tool for decision making in the selection of potential male sires.

Our results showed a large variation in mean testicular length and the presence of sperm at different ages. These findings are consistent with Galloway [7]. However, the number of observations in the upper end of the range is small; it would be beneficial for our model to include data from more animals at a greater age or with larger testicles (Fig. 2) but such animals were scarce in our study because of the owners’ decision when to castrate their alpacas. From our observations (Fig. 4) we would expect that animals with a testicular length ≥3.8 cm would be producing sperm (testicular length at which the median probability of sperm presence is ≥0.99); however, if it is crucial to know that there is no sperm production for management purposes, the threshold level for testicular length used to differentiate between sperm-producing and non-sperm producing animals should be ≤1.6 cm instead (testicular length at which the median probability of sperm presence is ≤0.01).

### Measurement of the testicles

Due to the small size of the alpaca testicles, their location very close to the abdomen and the difficulties in restraining the animals, only testicular length was measured in live animals in this study. According to Galloway [7] testicular length is a good measurement of testicle size in alpacas and it is easy to assess accurately. In bulls, measurement of the scrotal circumference is used for evaluating sperm-producing ability, because this is an objective measure, regardless of the operator, and has a strong correlation to the testicle size [18]. However, scrotal circumference is difficult to measure in alpacas due to their anatomical characteristics.

In our study, all the testicle measurements and BCS assessments were done by the same person, to minimize possible methodological errors. It should be noted that the testicular length taken from castrations or cadavers did not include the scrotal layers whereas, of necessity, the measurements on living animals did include the scrotum. Despite this difference in methodology, the measurements followed the same pattern.

### Puberty

Puberty is not a single point in time, but an ongoing process requiring endocrinological and behavioural components as well as anatomical and neurological development. In the literature there are different definitions of puberty, including the presence of fully developed genitalia, libido and a minimum concentration of sperm in the ejaculate. Galloway (personal communication, 2014) defines puberty in male alpacas as the moment when, for the first time, a male can mate a female and produce a pregnancy. For this to be possible, sperm production and good libido are necessary, and the penis should be completely free of adhesions. Although young males often begin to show interest in females and can display mounting behaviour around the age of one year, most of them are not able to carry out a mating at this age [19, 20]. Complete erection and intromission is only possible when the preputial attachments to the penis have totally disappeared [10]. The detachment seems to be affected positively by the level of testosterone [21]. Early detachment is a desirable genetic trait in young animals and therefore should be an important parameter in the evaluation of the breeding potential of males, together with large testis size [7, 10, 22] which is assumed to be directly related to sperm production, as in other ruminants [20]. In addition, it is important to know what testicular size should be considered as normal for the identification of pathological changes, e.g., hypoplasia or degeneration.

According to Tibary [23] the age at puberty varies depending on, among other factors, genetics, nutritional status, climate, and at what time of year the individual was born. Testicular volume may change according to environmental temperature [24]. Therefore, it would be interesting to do additional measurements of testicular length and body condition score in Sweden at different seasons of the year. Although studies suggest that female alpacas may show some seasonality in their willingness to mate, at least in the Peruvian Andes [25], there have not been any reports on the effect of season on male libido, as an indirect indicator of testosterone production. Moreover, if further studies on testicular measurements are to be made, it would be desirable to include a check for the breakdown of the preputial attachments, to provide additional information on the likelihood that mating can take place.

### Nutritional status/BCS

Peruvian alpacas have smaller testicles than Australian alpacas probably due to a poorer nutritional status [7]. Our results are in line with Galloway’s report, probably because alpaca husbandry in Sweden is likely to be more similar to Australian conditions than Peruvian conditions, especially in terms of nutrition and thus body weight. In this study, a significant positive effect of body condition on the testicle length increment rate was observed.

Fowler [26] indicates that overweight is probably a more common problem than emaciation in alpacas in the Western world, although a study carried out in Sweden by Björklund [2] found that emaciation can be a problem in weanling alpacas. It is worth mentioning that there are two different systems of production of South American camelids in the world: the traditional Andean herding strategies, in a pastoral economy in the dry highlands, with a high altitude grassland between 3000 and 4800 m above sea level, and the other system under very different and more favourable conditions, at a low altitude of no more than 800 m above sea level [27]. These husbandry conditions are likely to result in differing availability of nutrients and thus possibly the onset of puberty.

It is known that decreased nutrient intake delays the onset of puberty in bulls and that, conversely, a diet with high energy level produce a faster development of the testicles [28, 29]. However, there is little information regarding the effects of diet on reproductive function in male alpacas [6, 30]. It has been shown that certain parameters, including semen volume and sperm concentration as well as the biochemical composition of semen may vary depending on the feeding, but it is unclear if this has significance in practice [30].

### Breeding management

By detecting puberty in males as early as possible, owners are able to separate females and males to avoid undesirable matings, to use elite males strategically in breeding programs, and to castrate the ones that are not intended to be used as stud males.

When selecting males for breeding, owners should select early maturing males with large testicular size [7, 31]. Selecting for large testicular size, together with an evaluation of the sexual behavior, BCS and the detachment of preputial adherences, is likely to improve male reproductive efficiency.

Our study supports the recommendations of Vaughan [31]: Breeders/owners are encouraged to measure testicular length and BCS every 6 months, from 12 to 36 months of age, to assist with selection of potential male sires.

## Conclusions

If only one parameter is considered, testicular length is better than age alone to predict sperm production. However, the inclusion of BCS and age gives better predictability than a single parameter.

Our results suggests that animals with a testicular length ≥3.8 cm would be producing sperm; however, if it is crucial to know that there is no sperm production for management purposes, the threshold level for testicular length used to differentiate between sperm-producing and non-sperm producing animals should be ≤1.6 cm instead.

A combination of these parameters as a tool for decision making in the selection of potential male sires for animal husbandry under Swedish conditions is suggested.
